# Supplementary material for: High-recombining genomic regions affect demography inference based on ancestral recombination graphs
Source: Genetics. 2025 Jan 10;229(3):iyaf004. doi: 10.1093/genetics/iyaf004 (PMC11912872; doi:10.1093/genetics/iyaf004)
Supplement: iyaf004_Supplementary_Data [file iyaf004_supplementary_data.pdf]

Supplementary Information

High-recombining genomic regions affect demography inference based on ancestral recombination graphs

Jun Ishigohoka[1,*] Miriam Liedvogel[1,2,3,*]

[1]MPRG Behavioural Genomics, Max Planck Institute for Evolutionary Biology, Plön, Germany
[2]Institute of Avian Research, Wilhelmshaven, Germany
[3]Department of Biology and Environmental Sciences, Carl von Ossietzky Universität Oldenburg, Oldenburg, Germany

[*] Correspondence: Jun Ishigohoka <ishigohoka@evolbio.mpg.de>, Miriam Liedvogel <liedvogel@evolbio.mpg.de>

Table S1: Distributions of fitness effect used in forward simulation. f_i is the proportion of class i mutations in coding regions. The definition of each class is described in main text.

Scenario	f_0	f_1	f_2	f_3
DFE0	1	0	0	0
DFE1	0.7	0.1	0.1	0.1
DFE2	0.1	0.7	0.1	0.1
DFE3	0.1	0.1	0.7	0.1
DFE4	0.1	0.1	0.1	0.7

Table S2: Spearman correlation of true and estimated recombination rate by `LDhat`. Correlation could not be computed for $r/\mu = 0.1$ (represented as NA) because variance of recombination rate among windows of the true recombination map is 0.

r/μ	n	pop	Spearman's ρ
0.10	5	pop1	NA
0.10	5	pop2	NA
0.10	5	pop3	NA
0.10	10	pop1	NA
0.10	10	pop2	NA
0.10	10	pop3	NA
0.10	20	pop1	NA
0.10	20	pop2	NA
0.10	20	pop3	NA
0.25	5	pop1	0.816
0.25	5	pop2	0.817
0.25	5	pop3	0.811
0.25	10	pop1	0.832
0.25	10	pop2	0.834
0.25	10	pop3	0.829
0.25	20	pop1	0.836
0.25	20	pop2	0.838
0.25	20	pop3	0.836
1.00	5	pop1	0.833
1.00	5	pop2	0.835
1.00	5	pop3	0.828
1.00	10	pop1	0.838
1.00	10	pop2	0.838
1.00	10	pop3	0.835
1.00	20	pop1	0.839
1.00	20	pop2	0.839
1.00	20	pop3	0.838
4.00	5	pop1	0.835
4.00	5	pop2	0.836
4.00	5	pop3	0.831
4.00	10	pop1	0.839
4.00	10	pop2	0.839

r/μ	n	pop	Spearman's ρ
4.00	10	pop3	0.834
4.00	20	pop1	0.839
4.00	20	pop2	0.839
4.00	20	pop3	0.838
10.00	5	pop1	0.835
10.00	5	pop2	0.838
10.00	5	pop3	0.822
10.00	10	pop1	0.838
10.00	10	pop2	0.839
10.00	10	pop3	0.833
10.00	20	pop1	0.839
10.00	20	pop2	0.838
10.00	20	pop3	0.838

Table S3: Spearman correlation of true and estimated recombination rate by `pyrho`. Correlation could not be computed for $r/\mu = 0.1$ (represented as NA) because variance of recombination rate among windows of the true recombination map is 0.

r/μ	n	pop	Spearman's ρ
0.10	5	pop1	NA
0.10	5	pop2	NA
0.10	5	pop3	NA
0.10	10	pop1	NA
0.10	10	pop2	NA
0.10	10	pop3	NA
0.10	20	pop1	NA
0.10	20	pop2	NA
0.10	20	pop3	NA
0.25	5	pop1	0.962
0.25	5	pop2	0.963
0.25	5	pop3	0.965
0.25	10	pop1	0.986
0.25	10	pop2	0.991
0.25	10	pop3	0.981
0.25	20	pop1	0.991
0.25	20	pop2	0.992
0.25	20	pop3	0.990
1.00	5	pop1	0.978
1.00	5	pop2	0.968
1.00	5	pop3	0.981
1.00	10	pop1	0.993
1.00	10	pop2	0.994
1.00	10	pop3	0.989
1.00	20	pop1	0.995
1.00	20	pop2	0.994

r/μ	n	pop	Spearman's ρ
1.00	20	pop3	0.992
4.00	5	pop1	0.993
4.00	5	pop2	0.978
4.00	5	pop3	0.989
4.00	10	pop1	0.991
4.00	10	pop2	0.993
4.00	10	pop3	0.986
4.00	20	pop1	0.994
4.00	20	pop2	0.992
4.00	20	pop3	0.989
10.00	5	pop1	0.972
10.00	5	pop2	0.965
10.00	5	pop3	0.977
10.00	10	pop1	0.992
10.00	10	pop2	0.989
10.00	10	pop3	0.987
10.00	20	pop1	0.995
10.00	20	pop2	0.995
10.00	20	pop3	0.992

Table S4: Spearman correlation of true and estimated recombination rate by `iSMC`. Correlation could not be computed for $r/\mu = 0.1$ (represented as NA) because variance of recombination rate among windows of the true recombination map is 0.

r/μ	n	cor	Spearman's ρ
0.10	1	pop1	0.000
0.10	1	pop2	0.000
0.10	1	pop3	0.000
0.25	1	pop1	0.839
0.25	1	pop2	0.843
0.25	1	pop3	0.839
1.00	1	pop1	0.839
1.00	1	pop2	0.839
1.00	1	pop3	0.839
4.00	1	pop1	0.838
4.00	1	pop2	0.836
4.00	1	pop3	0.838
10.00	1	pop1	0.838
10.00	1	pop2	0.838
10.00	1	pop3	0.837

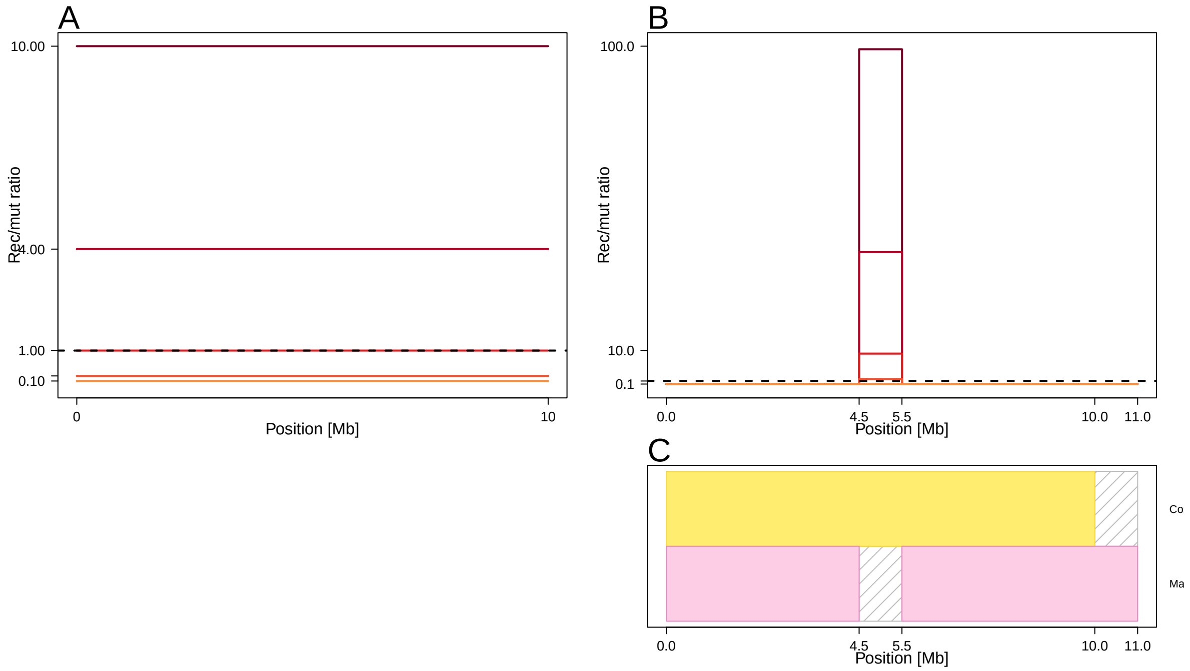

Figure S1: Two scenarios with different sets of recombination landscapes. A. Recombination maps of the "narrow high-rec." scenarios. **B.** Recombination maps of the "uniform" scenarios **C.** Two settings of demography inference masking different parts of the chromosomes for the narrow high-rec scenario (Yellow shades depict genomic regions used in demography inference. Gray shades depict regions masked from demography inference). We asked whether the presence of the high-recombining region (i.e. in the middle of the chromosome) affects demography inference and whether masking them improves demography inference. We applied methods of demography inference on the simulated data using different parts of the chromosome: either including the high-recombining regions ("control", top) or masking the 1 Mb of high-recombining regions (bottom). To control for the amount of sequences used in the inferences, we applied 1 Mb mask outside the high-recombining regions in the first setting (top).

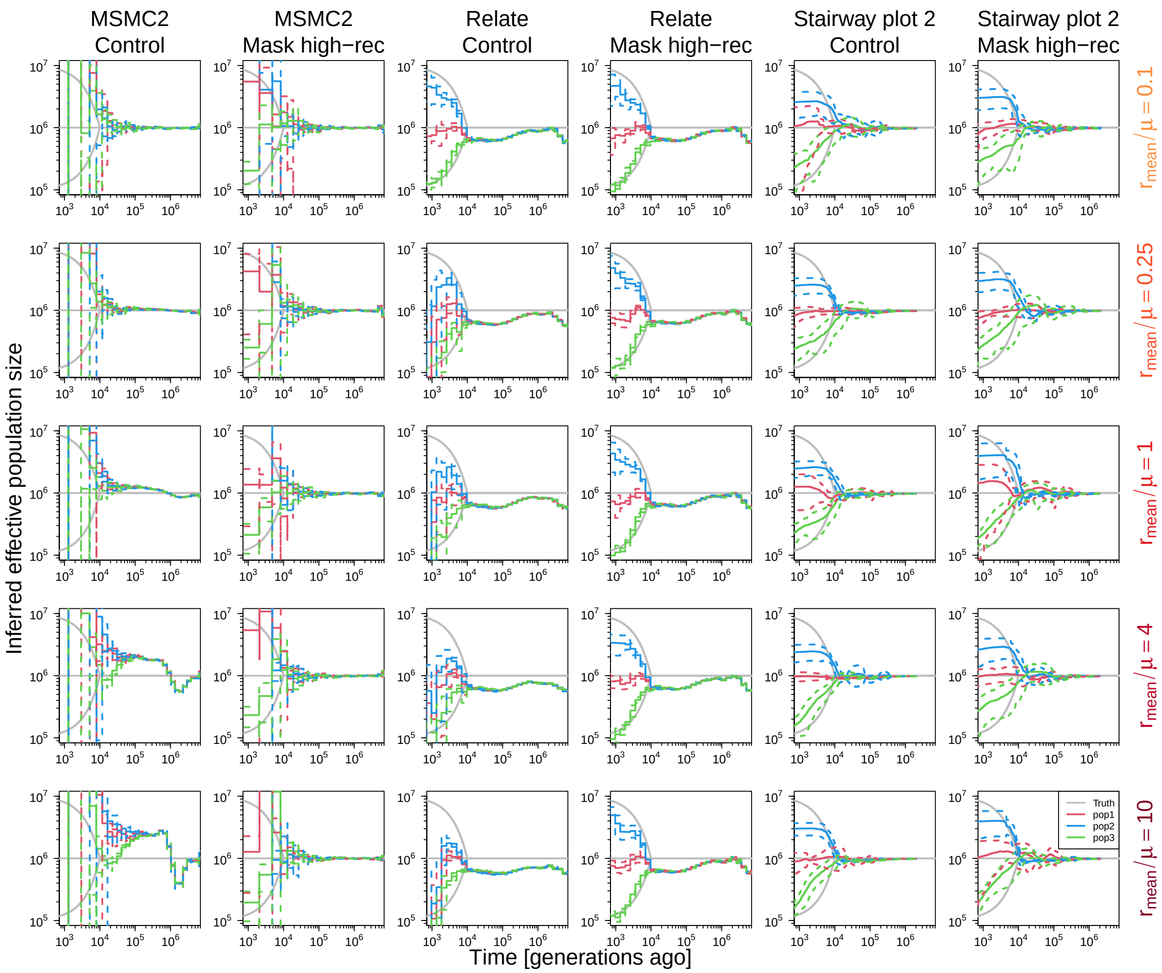

Figure S2: Inference of historical effective population size with stepwise recombination landscapes under demography model 1 ($N_{anc} = 1,000,000$). The inferences by `MSMC2` and `Relate` without masking high-recombining regions show that presence of high-recombining regions impacts ARG-based methods. Masking the high-recombining regions eliminated this effect. `Stairwayplot 2` is not affected by the presence of high-recombining regions. In each panel, gray lines depict the simulated truths (as in Fig. 2a), solid and dashed coloured lines depict the mean and mean ± SD of the inferences (see Materials and Methods for details on how replicates were treated).

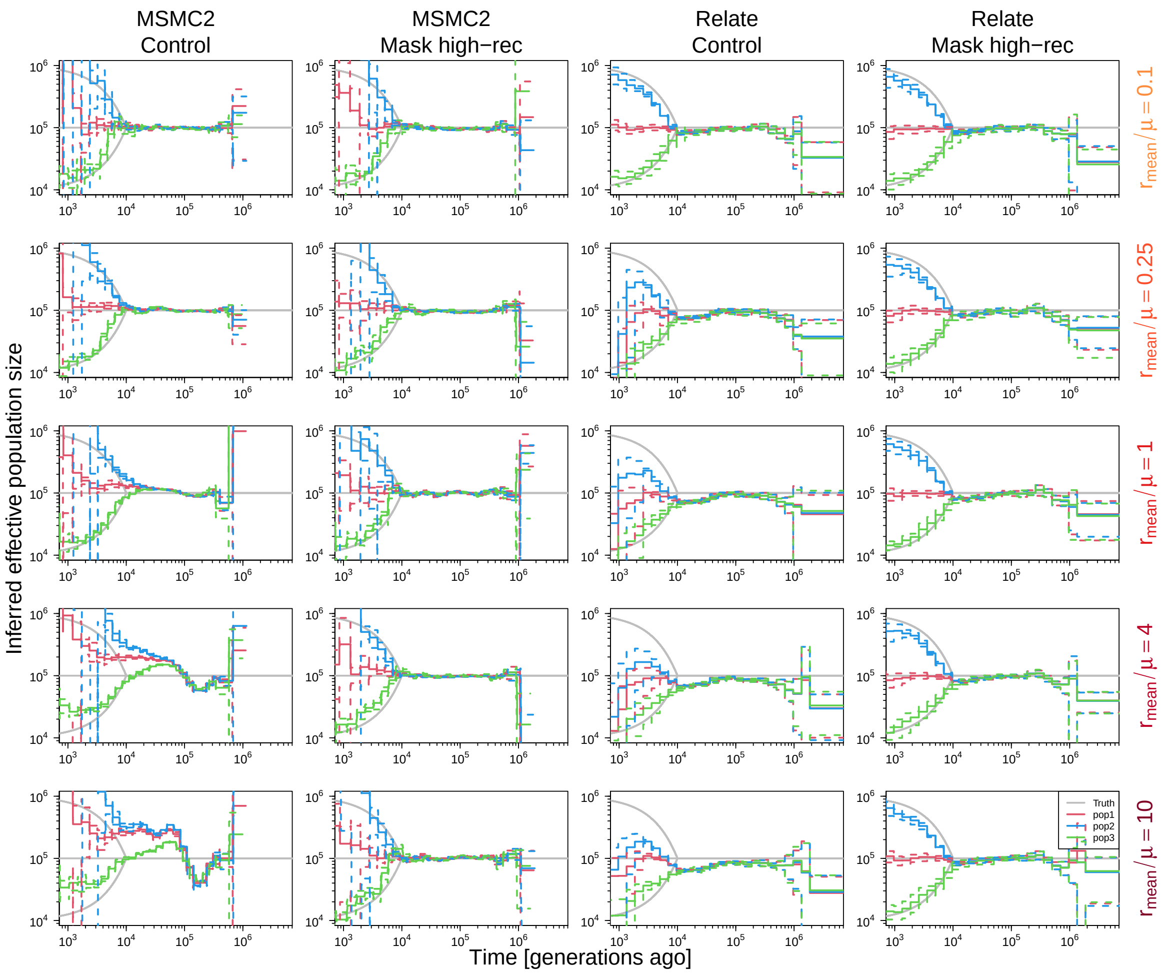

Figure S3: Inference of historical effective population size with stepwise recombination landscapes under demography model 2 ($N_{anc} = 100,000$). The inferences by `MSMC2` and `Relate` without masking high-recombining regions show that presence of high-recombining regions impacts ARG-based methods. Masking the high-recombining regions eliminated this effect. In each panel, gray lines depict the simulated truths (as in Fig. 2a), solid and dashed coloured lines depict the mean and mean ± SD of the inferences (see Materials and Methods for details on how replicates were treated).

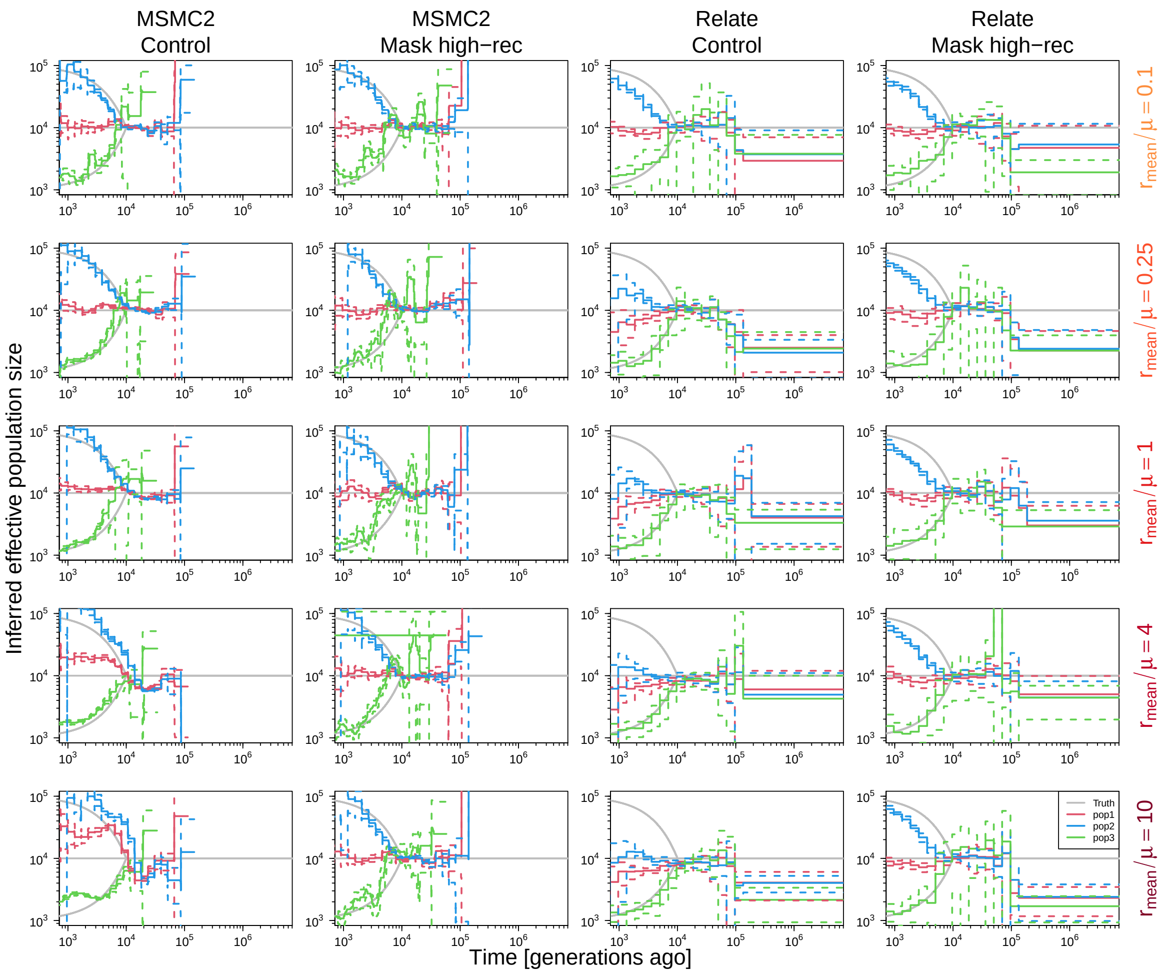

Figure S4: Inference of historical effective population size with stepwise recombination landscapes under demography model 3 ($N_{anc} = 10,000$). The inferences by `MSMC2` and `Relate` without masking high-recombining regions show that presence of high-recombining regions impacts ARG-based methods. Masking the high-recombining regions eliminated this effect. In each panel, gray lines depict the simulated truths (as in Fig. 2a), solid and dashed coloured lines depict the mean and mean ± SD of the inferences (see Materials and Methods for details on how replicates were treated).

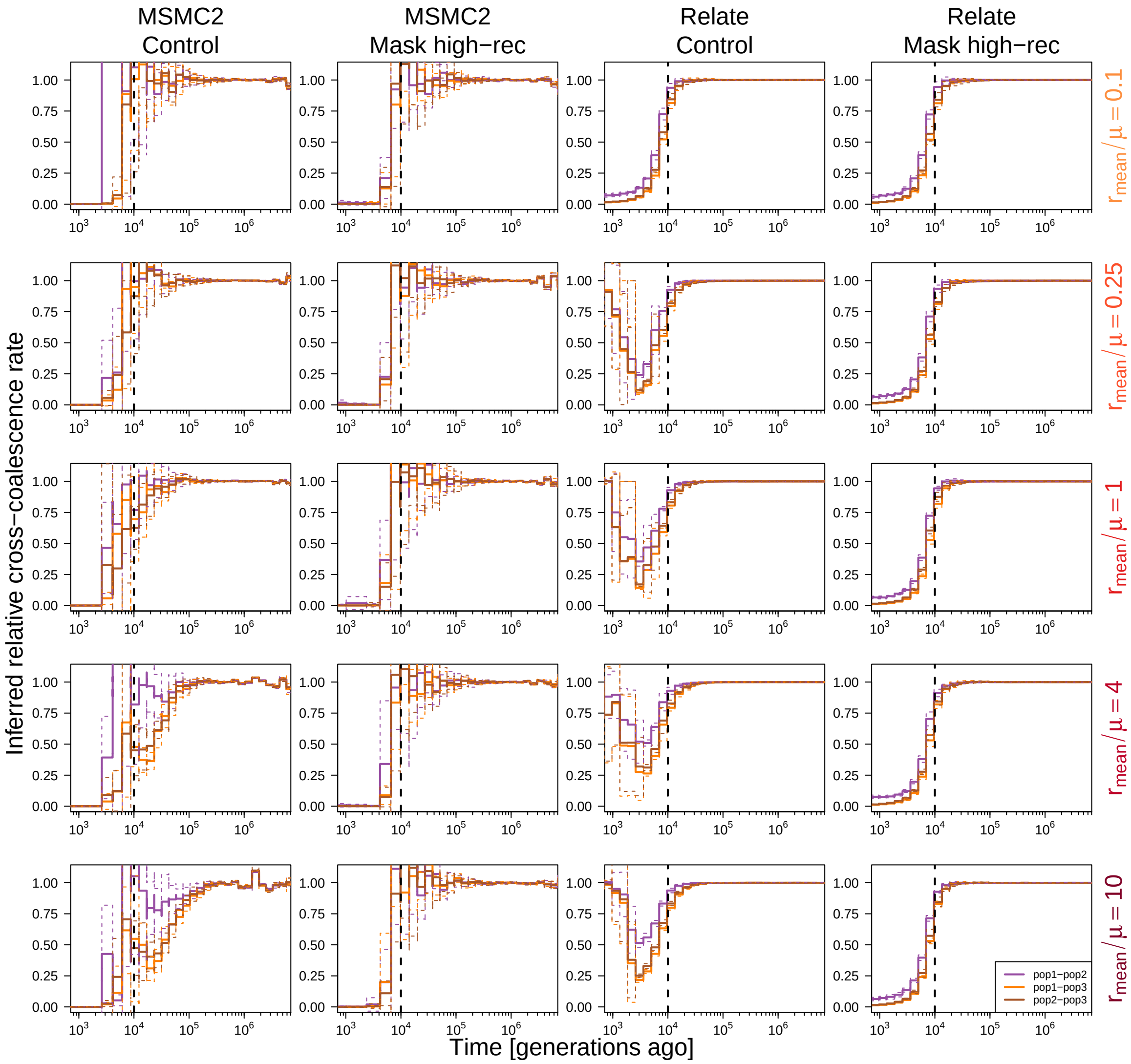

Figure S5: Inference of relative cross-coalescence rate with stepwise recombination landscapes under demography model 1 ($N_{anc} = 1,000,000$). Vertical dotted lines depict the true split time. Coloured lines depict inferred rCCR for pairs of populations. Three colours indicate three pairs of populations. `MSMC2` (left two columns). Solid lines depict mean of inferences of the ten down-samples, and dotted lines depict mean ± SD. The results show that presence of high-recombining regions affects inference of population splits, and removing the high-recombining regions improves it. `Relate` (right two columns). Solid lines depict mean of inferences of the ten replicates, and dotted lines depict mean ± SD. The results show that presence of high-recombining regions affects inference of population splits, and removing the high-recombining regions improves it.

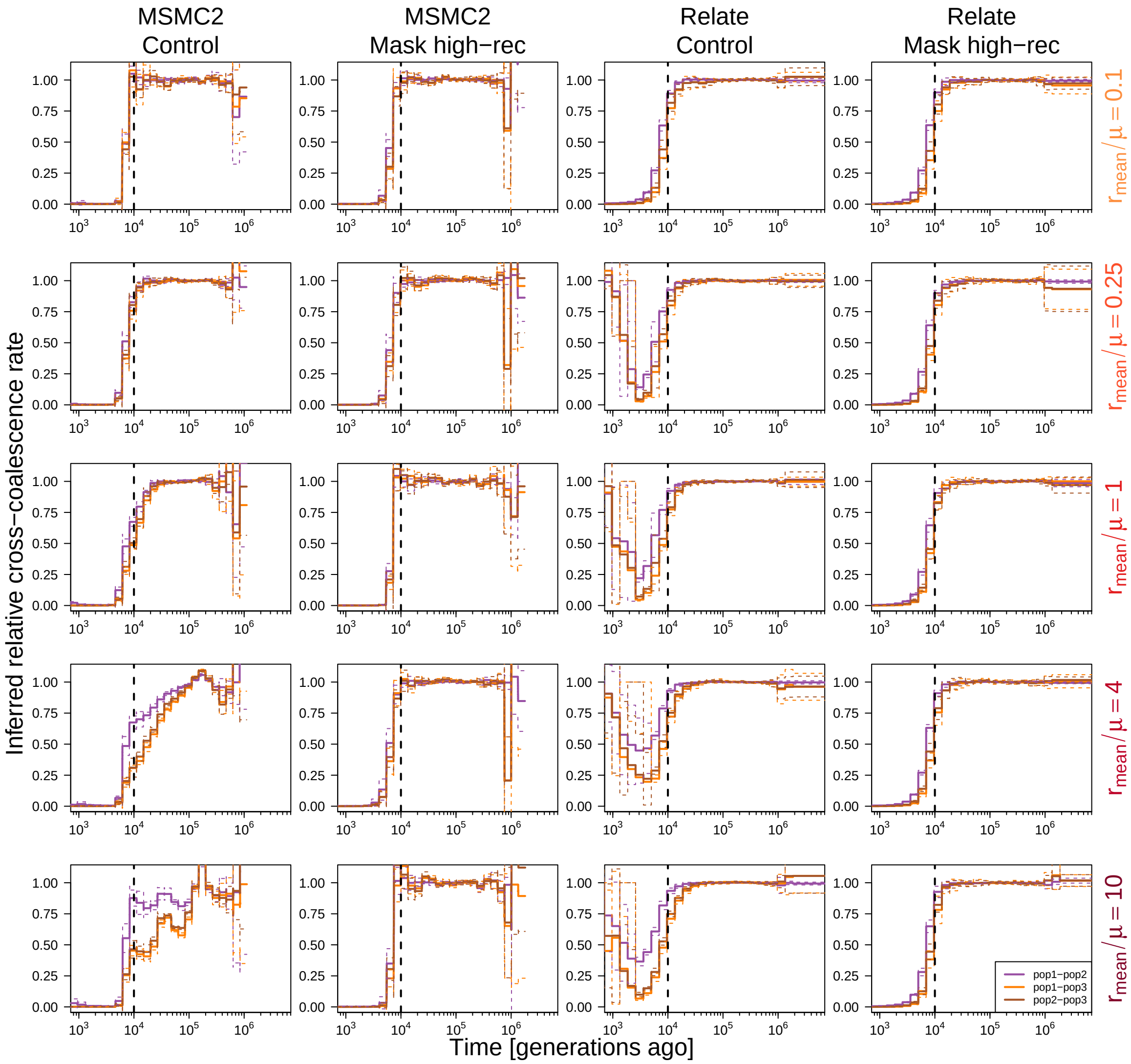

Figure S6: Inference of relative cross-coalescence rate with stepwise recombination landscapes under demography model 2 ($N_{anc} = 100,000$)**.** Vertical dotted lines depict the true split time. Coloured lines depict inferred rCCR for pairs of populations. Three colours indicate three pairs of populations. `MSMC2` (left two columns). Solid lines depict mean of inferences of the ten down-samples, and dotted lines depict mean ± SD. The results show that presence of high-recombining regions affects inference of population splits, and removing the high-recombining regions improves it. `Relate` (right two columns). Solid lines depict mean of inferences of the ten replicates, and dotted lines depict mean ± SD. The results show that presence of high-recombining regions affects inference of population splits, and removing the high-recombining regions improves it.

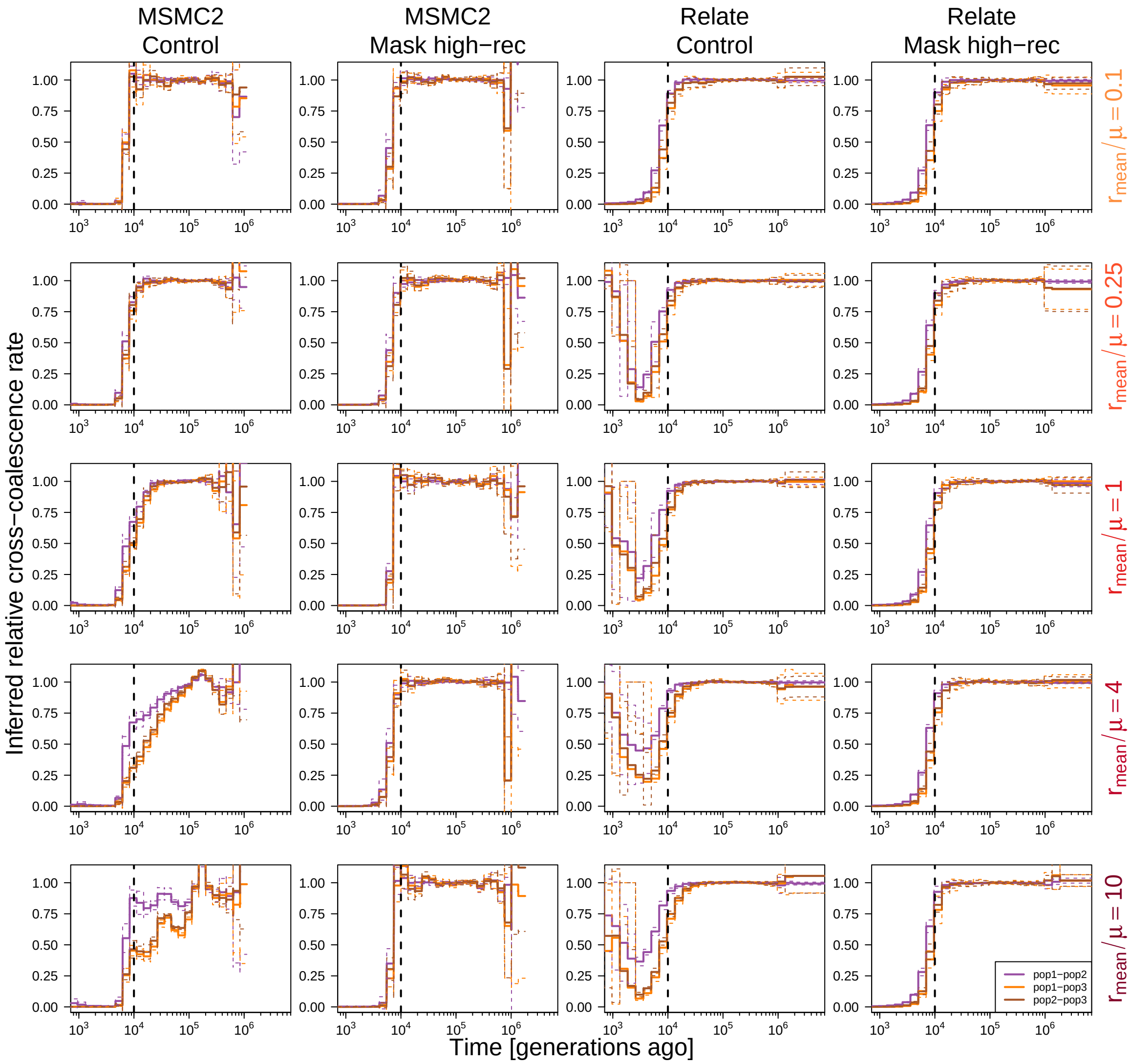

Figure S7: Inference of relative cross-coalescence rate with stepwise recombination landscapes under demography model 3 ($N_{anc} = 10,000$). Vertical dotted lines depict the true split time. Coloured lines depict inferred rCCR for pairs of populations. Three colours indicate three pairs of populations. `MSMC2` (left two columns). Solid lines depict mean of inferences of the ten down-samples, and dotted lines depict mean ± SD. The results show that presence of high-recombining regions affects inference of population splits, and removing the high-recombining regions improves it. `Relate` (right two columns). Solid lines depict mean of inferences of the ten replicates, and dotted lines depict mean ± SD. The results show that presence of high-recombining regions affects inference of population splits, and removing the high-recombining regions improves it.

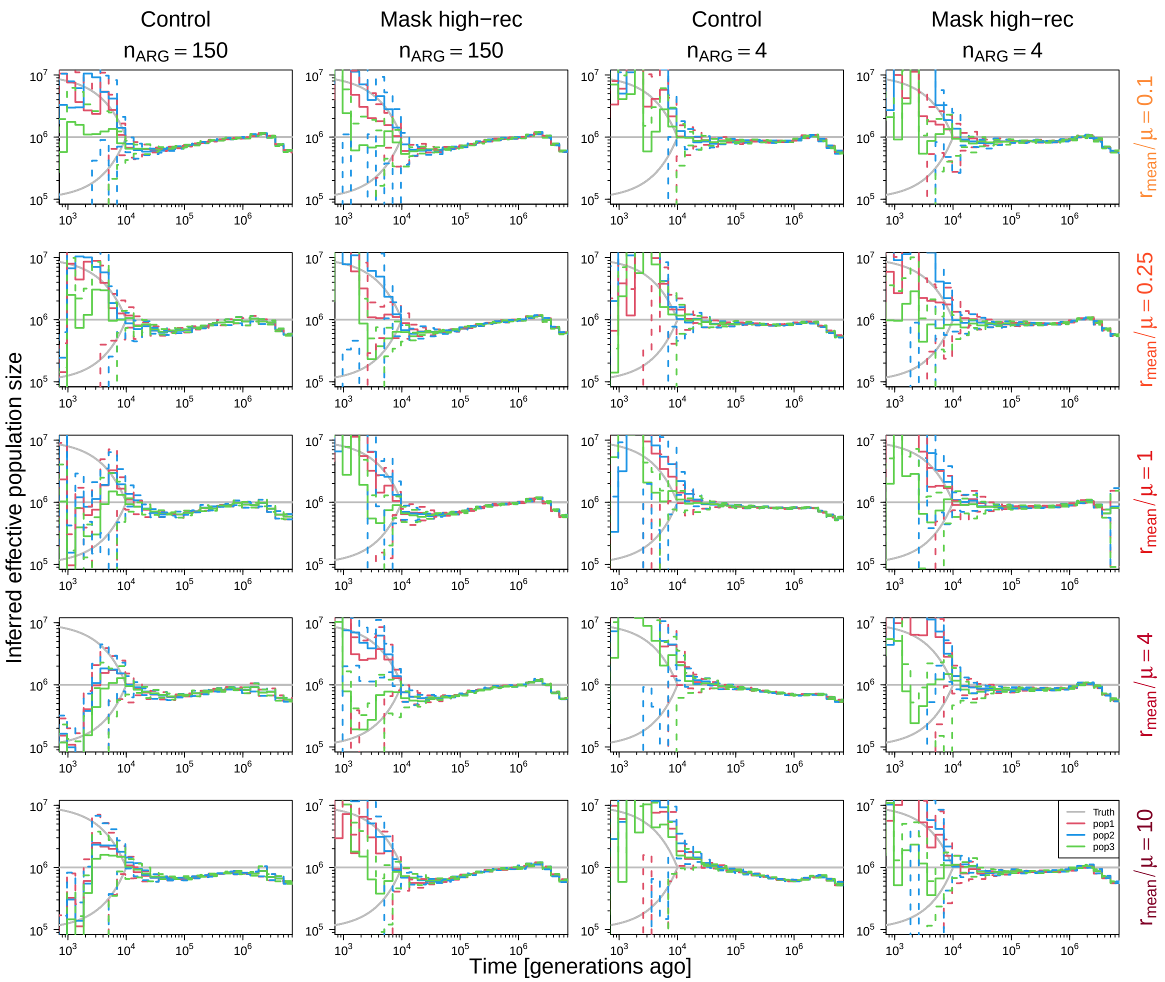

Figure S8: Inference of effective population size by `Relate` with stepwise recombination landscapes under demography model 1 ($N_{anc} = 1,000,000$) with sample size comparable to `MSMC2`. In the first and second columns, ARG was inferred using 150 diploids, then four diploids per population were used for demography inference. In the third and fourth columns, ARG was inferred using 4 diploids per population (12 diploids in total), and demography was inferred using all 12 diploids. Solid lines depict mean of inferences of the ten replicates, and dotted lines depict mean ± SD.

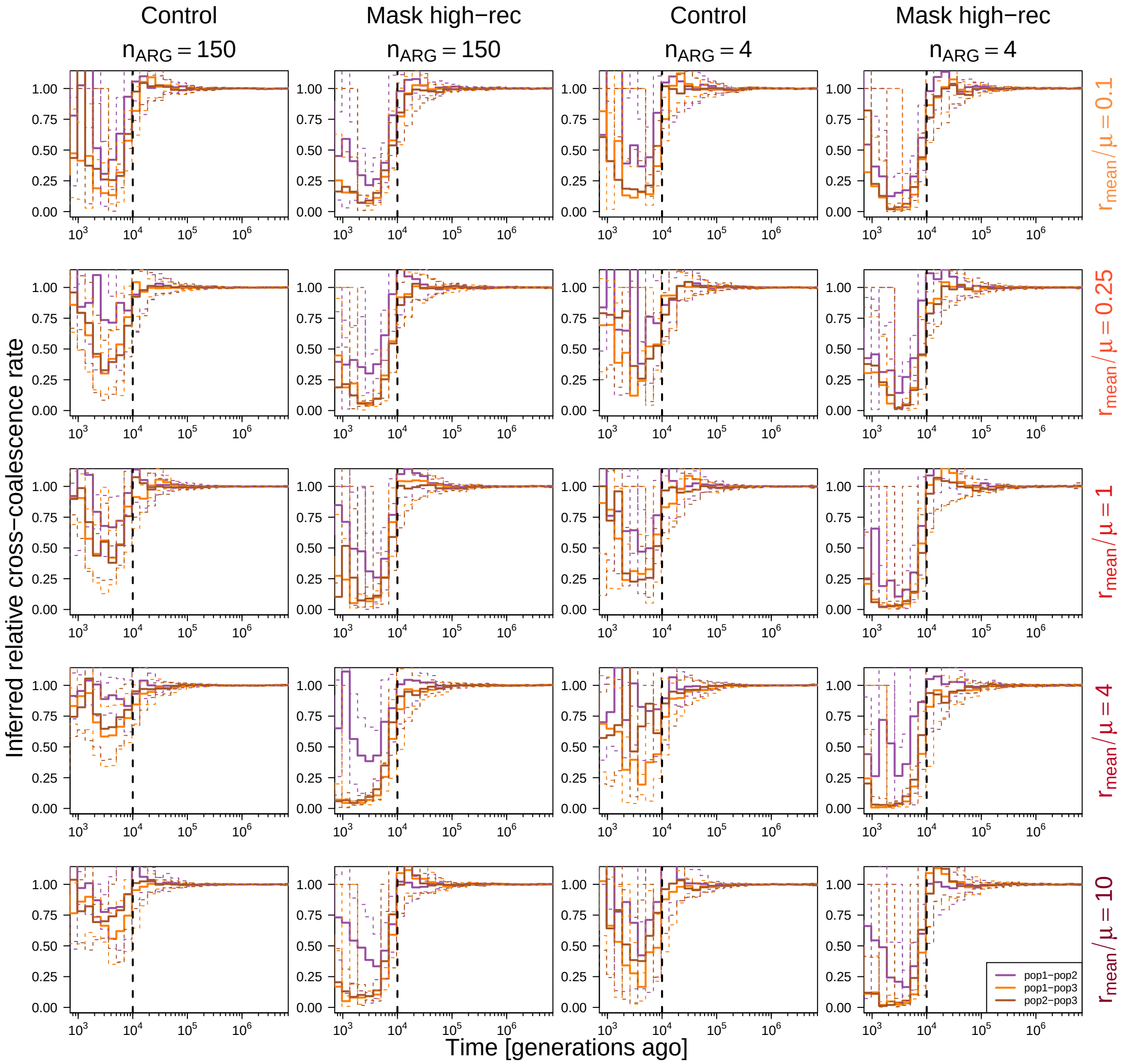

Figure S9: Inference of effective population size by `Relate` with stepwise recombination landscapes under demography model 1 ($N_{anc} = 1,000,000$) with sample size comparable to `MSMC2`. In the first and second columns, ARG was inferred using 150 diploids, then four diploids per population were used for demography inference. In the third and fourth columns, ARG was inferred using 4 diploids per population (12 diploids in total), and demography was inferred using all 12 diploids. Solid lines depict mean of inferences of the ten replicates, and dotted lines depict mean ± SD.

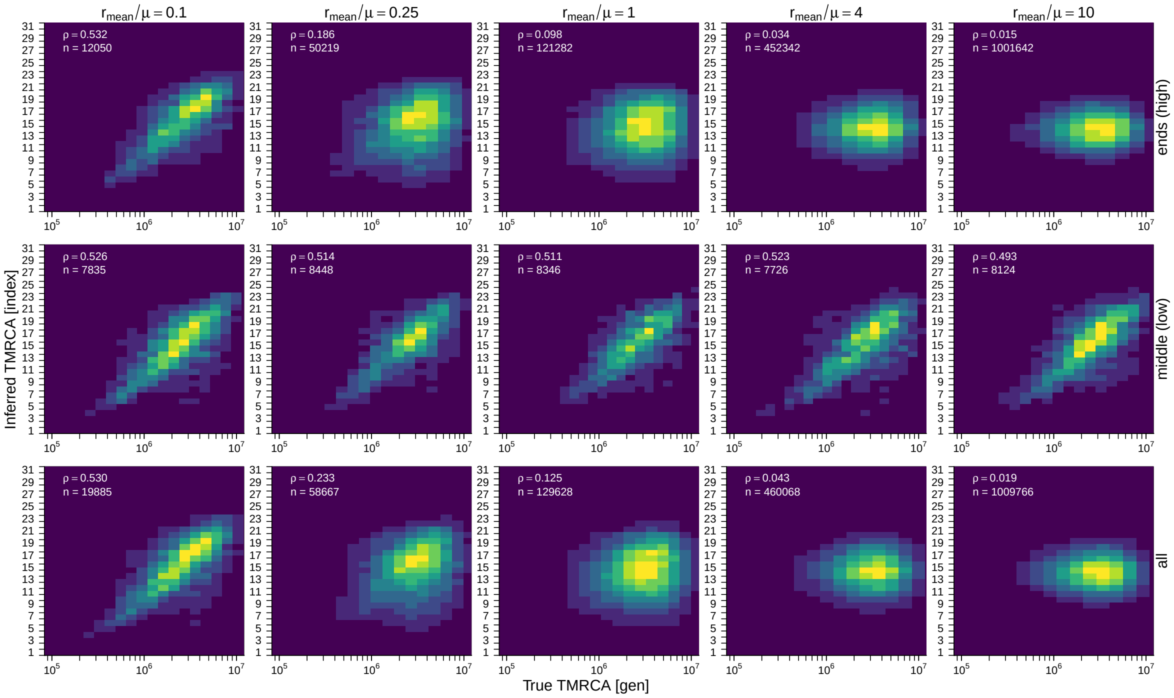

Figure S10: Inference of representation of genealogies by MSMC2 is affected specifically within high-recombining regions. Coalescence time between two sequences (haplotypes 0 and 1 (pop1)) were compared between the simulated truth (x axis) and inference by MSMC2 (the discretised epoch with the highest likelihood, y axis). Top row: two 3-Mb intervals in both ends of the simulated chromosome with elevated recombination rates. Middle row: a 6-Mb intervals in the centre of the chromosome with recombination rate 1/10 of mutation rate. Bottom row: both two 3-Mb ends and 6-Mb middle intervals. Five columns correspond to different recombination maps shown in Fig. 2. n is the number of genomic intervals whose boundaries are defined by either a true genealogy or an HMM window in MSMC2. ρ is the Spearman's coefficient of correlation.

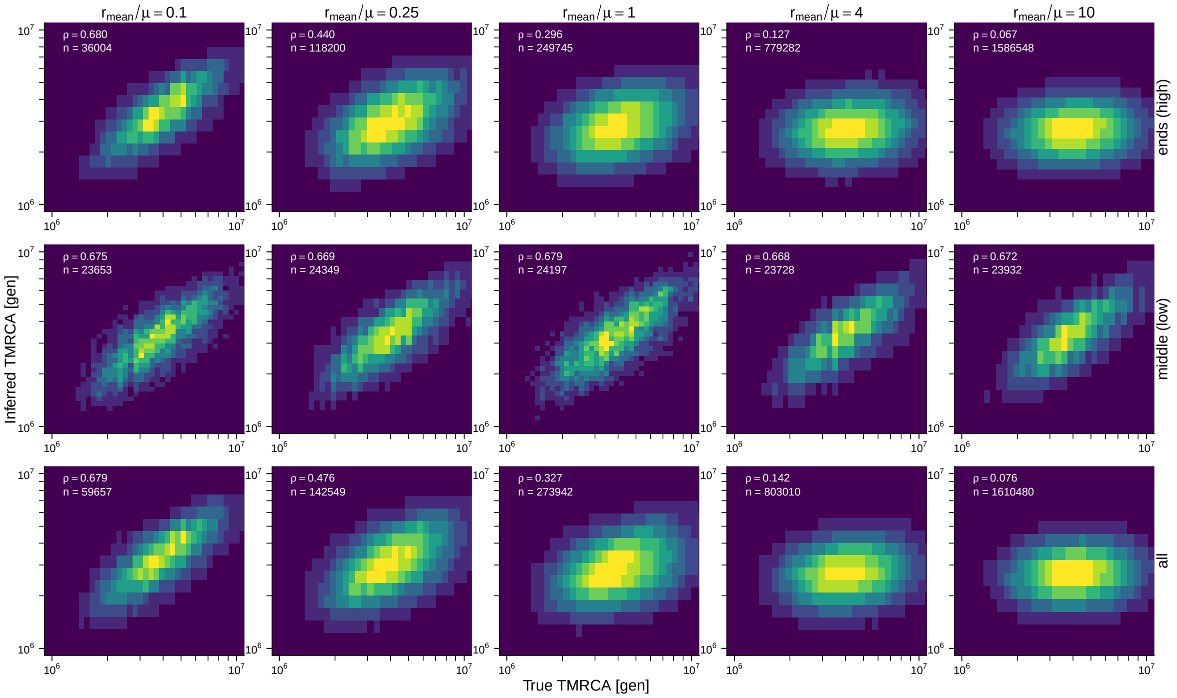

Figure S11: Inference of genealogies by `Relate` is affected specifically within high-recombining regions. Time to the final coalescence (tree height) of genealogies of 300 sequences (50 diploids per population) were compared between the simulated truth (x axis) and inference by `Relate` (y axis). Top row: two 3-Mb intervals in both ends of the simulated chromosome with elevated recombination rates. Middle row: a 6-Mb intervals in the centre of the chromosome with recombination rate 1/10 of mutation rate. Bottom row: both two 3-Mb ends and 6-Mb middle intervals. n is the number of genomic intervals whose boundaries are defined by either a true or inferred genealogy. ρ is the Spearman's coefficient of correlation.

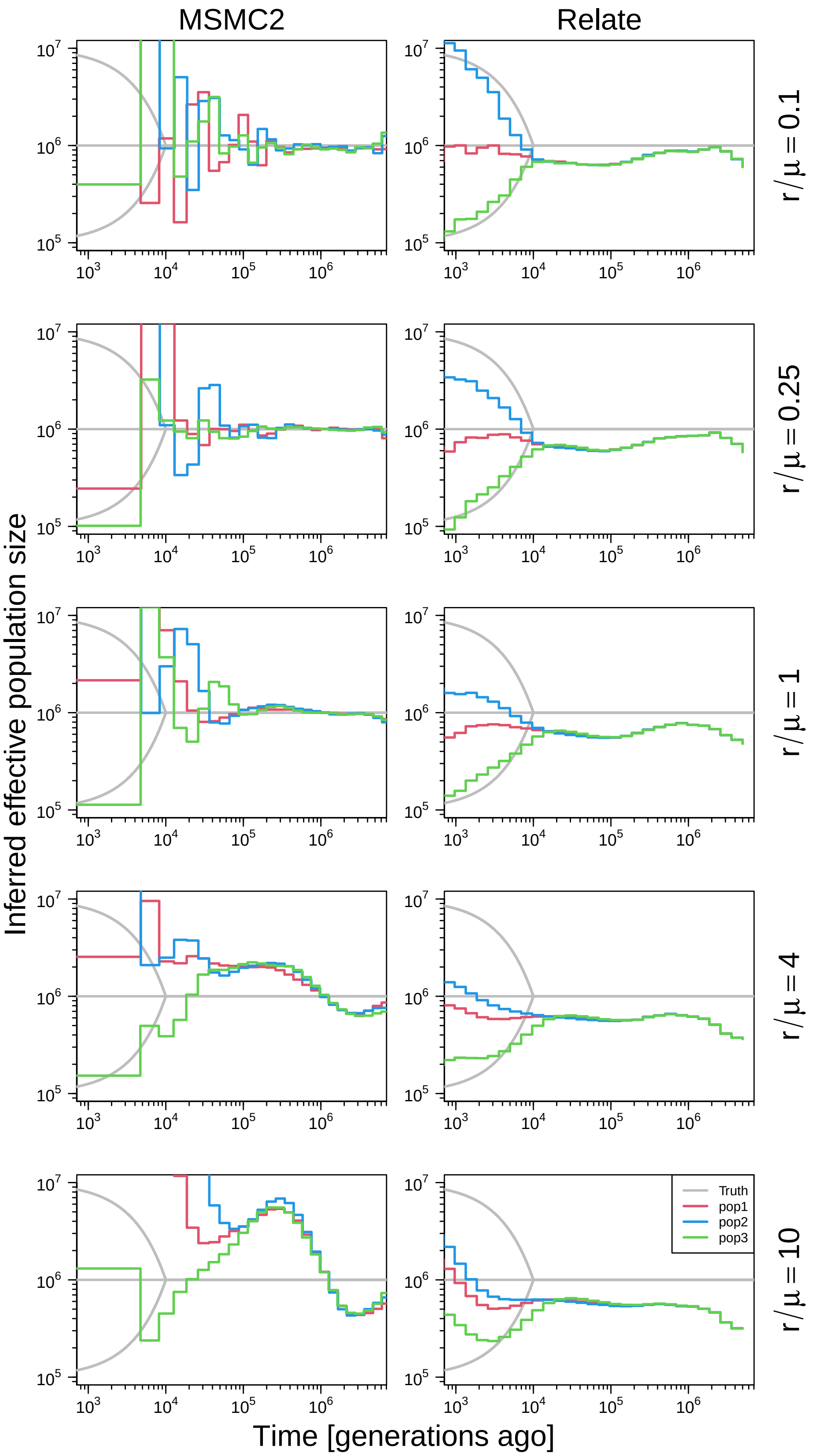

Figure S12: Inference of effective population size by `MSMC2` and `Relate` under the uniform recombination landscape scenarios. The effect of recombination rate is qualitatively consistent with the results with stepwise recombination landscapes (Fig. 3, Fig S2) but more pronounced.)

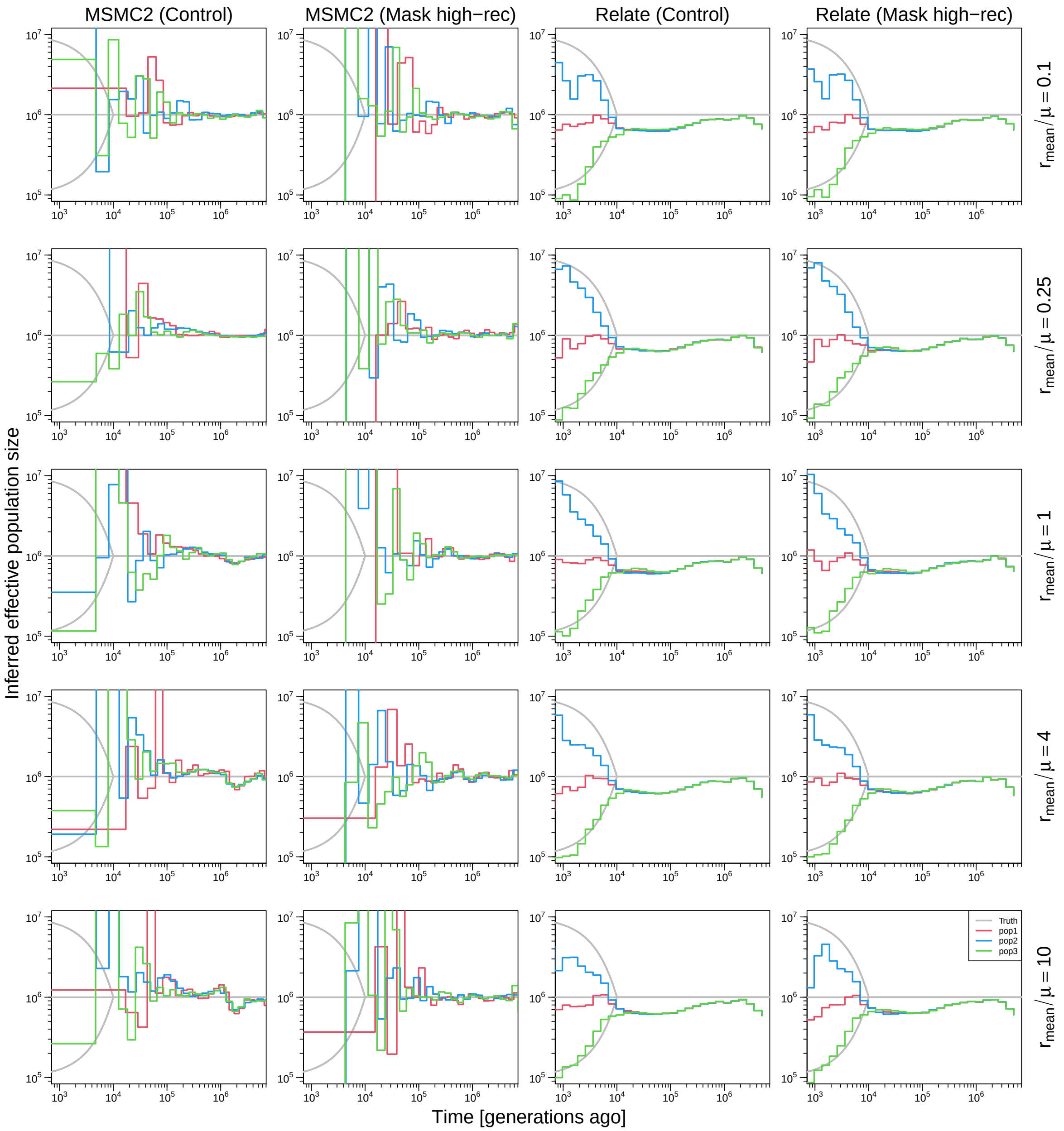

Figure S13: Inference of effective population size by MSMC2 and Relate under the recombination landscape scenarios with a single narrow high-recombining region. In MSMC2, the effect of the high-recombining region is qualitatively consistent with the results with stepwise recombination landscapes (Fig. 3, Fig S2) but less pronounced.) Relate, is robust to the narrow high-recombining region.

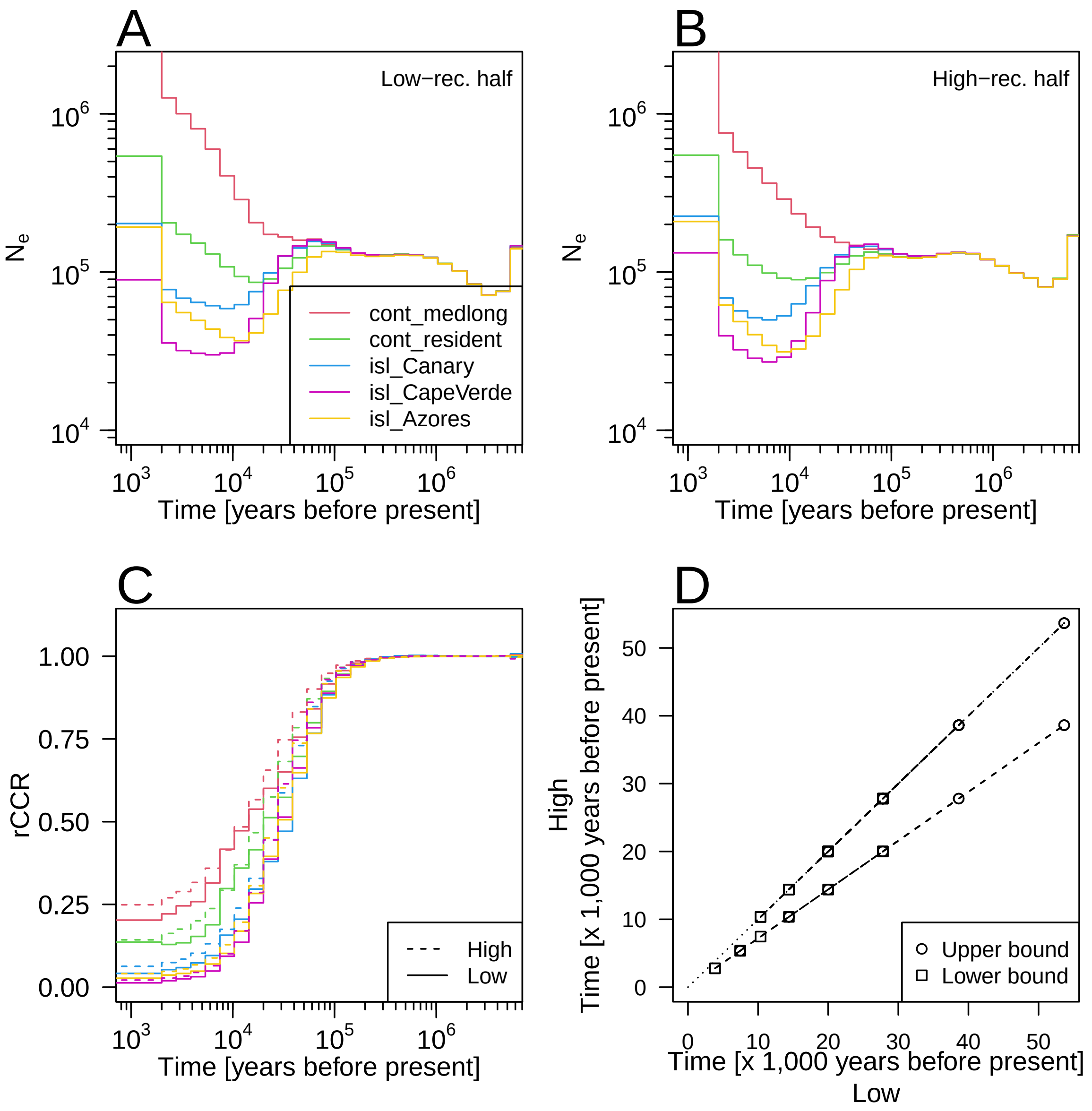

Figure S14: Demography inference by `Relate` with lower- and higher-recombining halves of blackcap genomes. A, B. Inference of historical effective population size. **C**. Inference of relative cross-coalescence rate (rCCR) between Azores population and each of all other four populations in **A** and **B** using the lower (solid lines) and the higher (dotted lines) half of the genome based on local recombination rates. **D**. Comparison of split times inferred by `Relate` using the lower higher halves of the genome based on recombination rates. Segments represent inference between 45 pairs of 10 populations. Two ends of a segment represent the lower and upper boundaries of two consecutive discretised epochs between which rCCR crosses the threshold of 0.5.

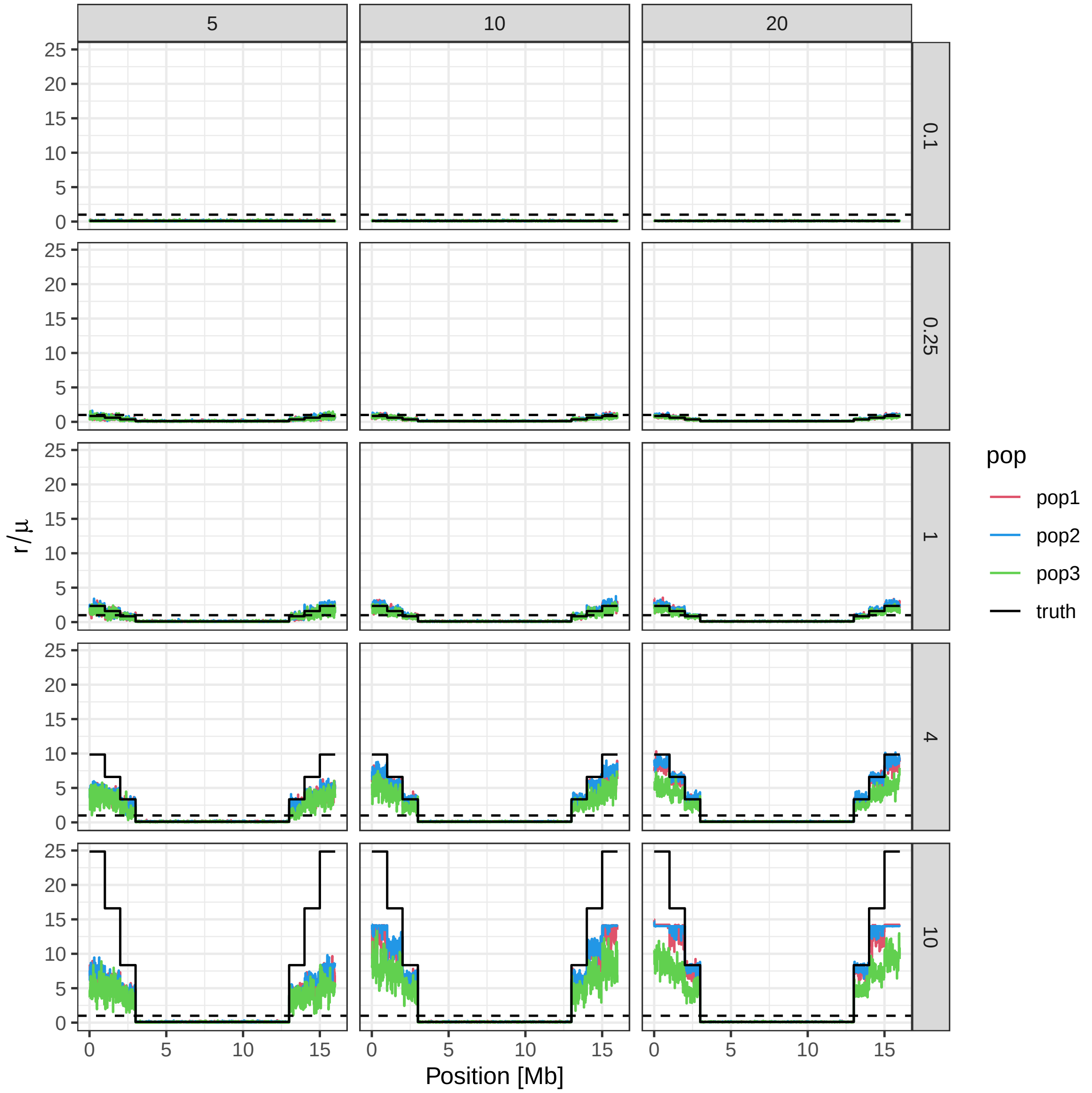

Figure S15: Recombination map inference using `LDhat`. Rows and columns represent recombination rate at high-recombining regions and sample size per population. The horizontal dotted line shows the level where $r = \mu$.

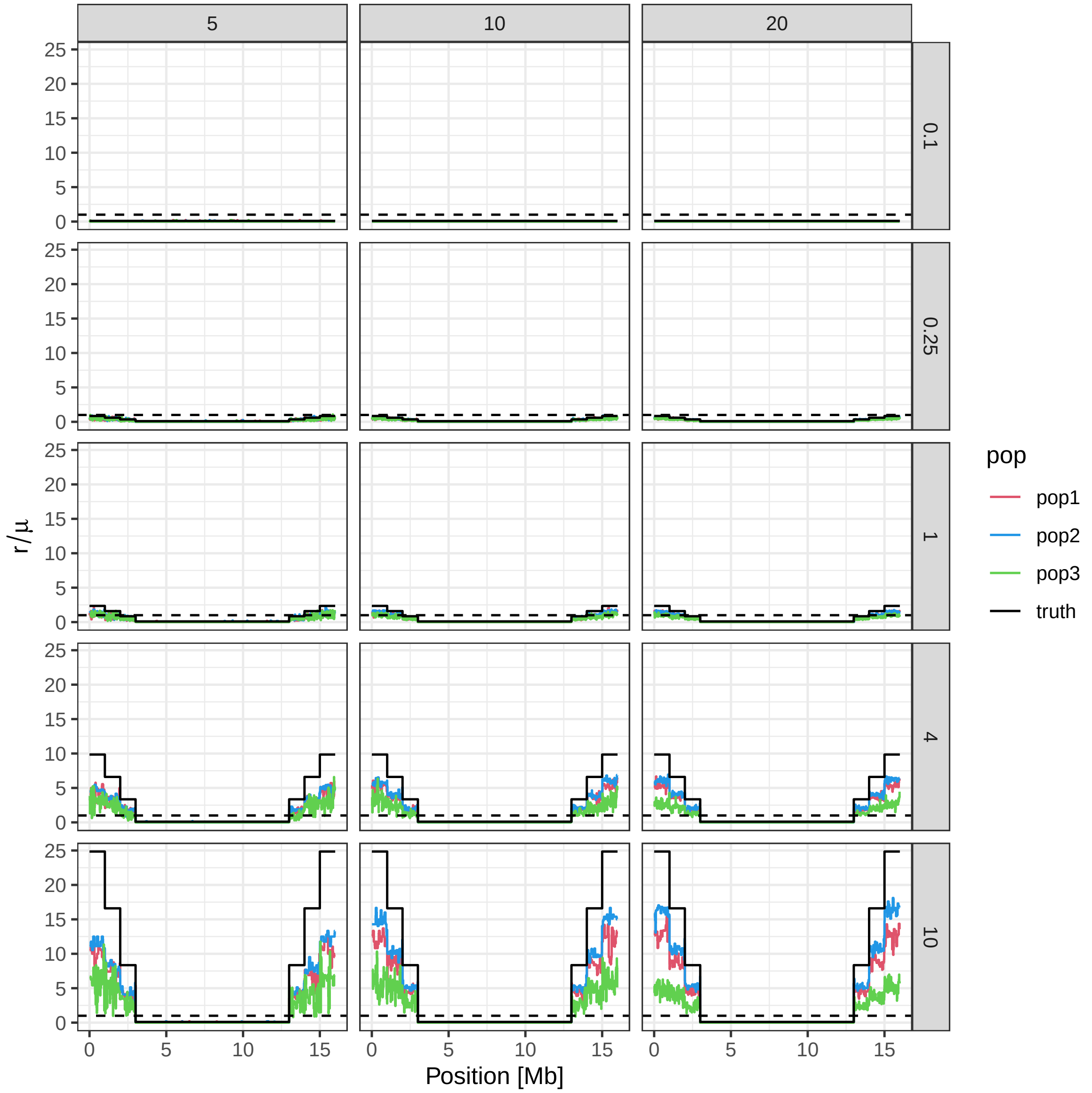

Figure S16: Recombination map inference using `pyrho`. Rows and columns represent recombination rate at high-recombining regions and sample size per population. The horizontal dotted line shows the level where $r = \mu$.

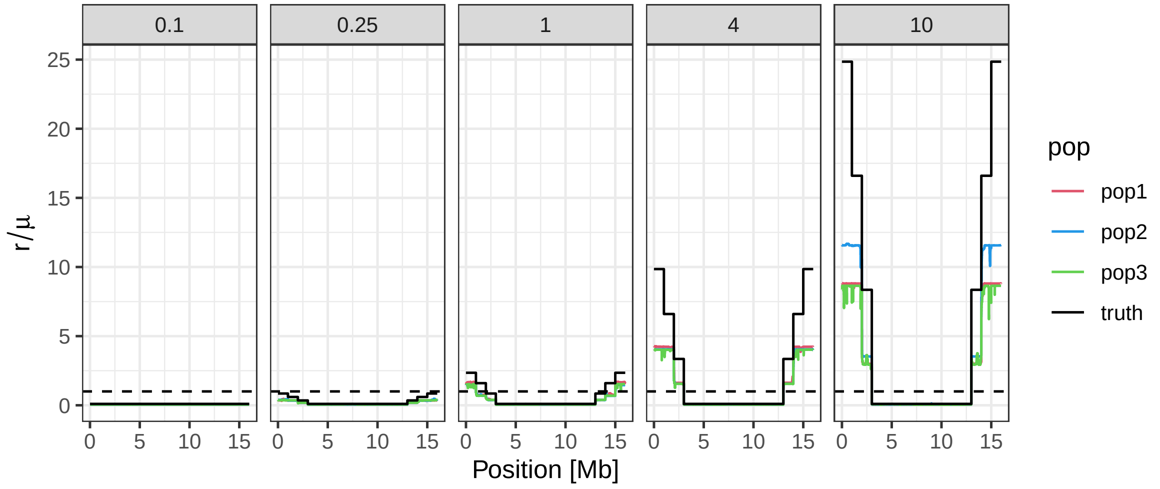

Figure S17: Recombination map inference using `iSMC`. Columns represent scenarios with different recombination rate at high-recombining regions. The plotted r/μ is based on jointly estimated ρ and θ. The horizontal dotted line shows the level where $r = \mu$.

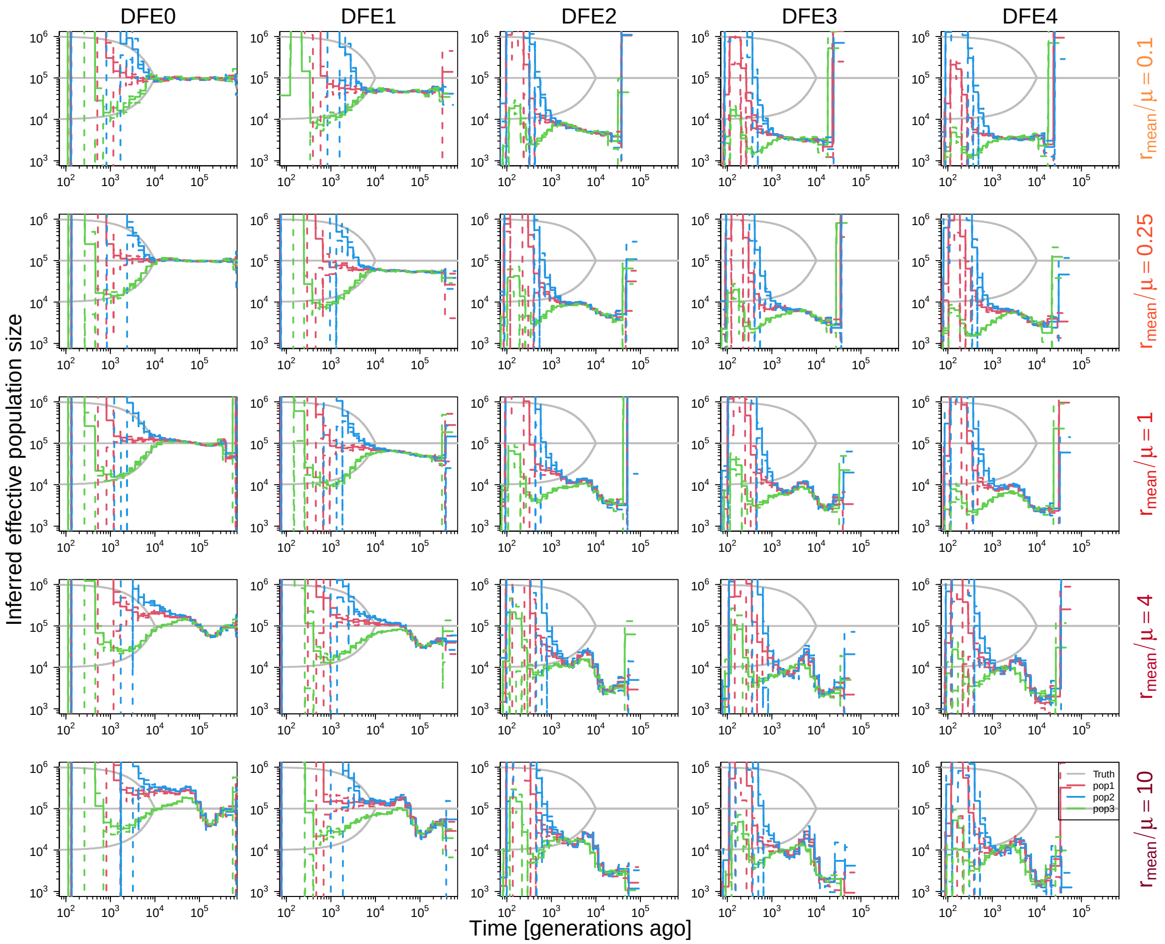

Figure S18: Inference of effective population size by MSMC2 with background selection under control condition (not masking high-recombining regions). Columns represent different DFEs from neutral to strong background selection. Rows represent the stepwise recombination maps. The results show that background selection affects the scaling of inferred demography both in effective population size and time.

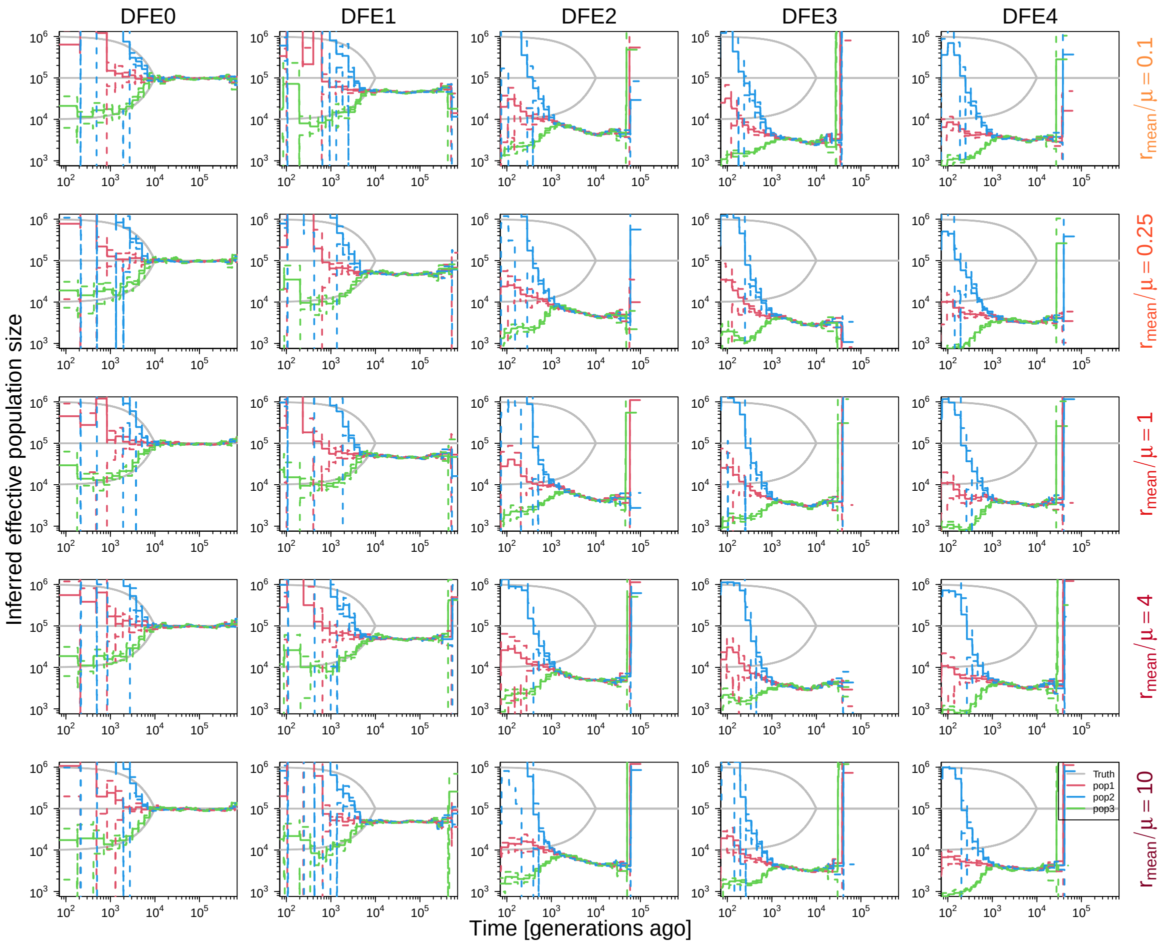

Figure S19: Inference of effective population size by MSMC2 with background selection masking high-recombining regions. Columns represent different DFEs from neutral to strong background selection. Rows represent the stepwise recombination maps. The results show that while masking high-recombining regions eliminated the effect of high-recombining regions on the shape of demography trajectory, it does not remove the effect by background selection.

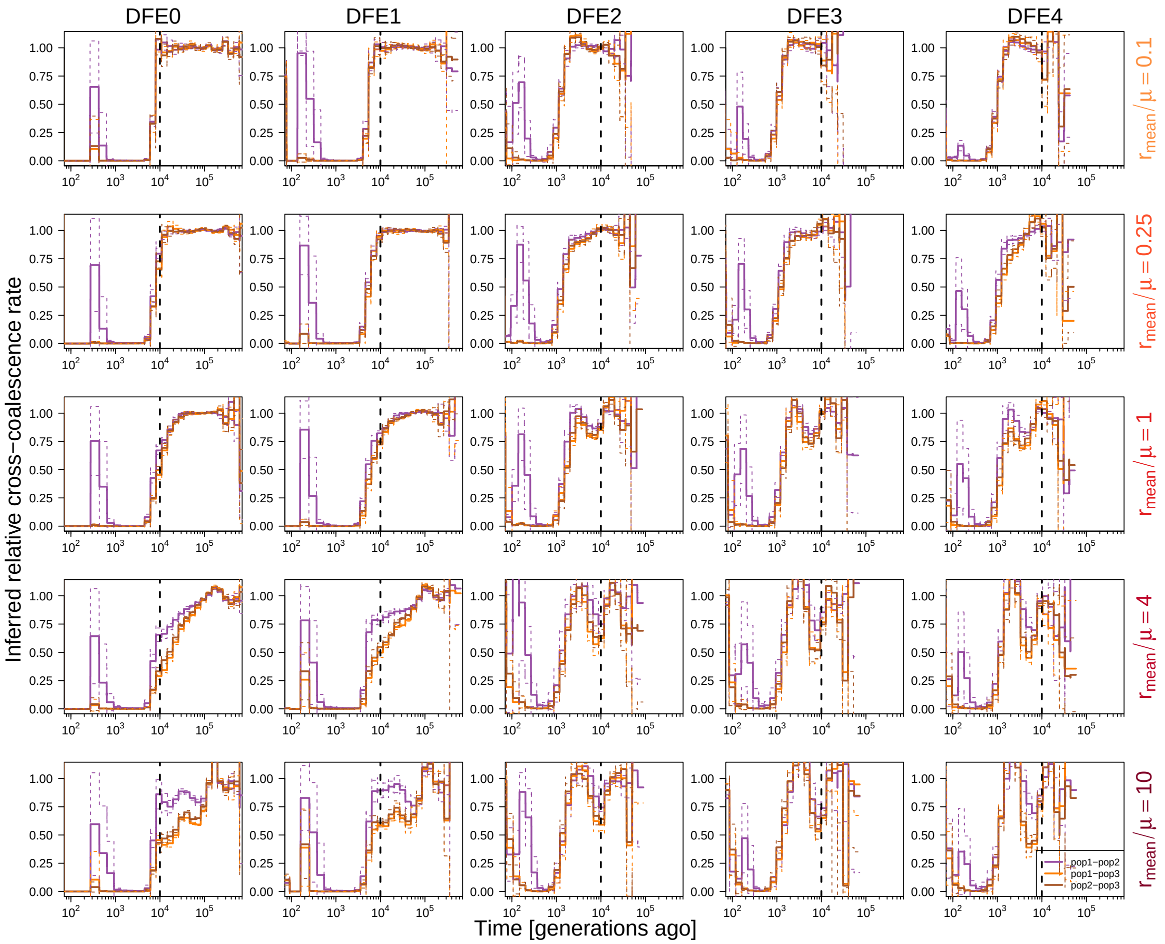

Figure S20: Inference of relative cross-coalescence rate by `MSMC2` with background selection under control condition (not masking high-recombining regions). Columns represent different DFEs from neutral to strong background selection. Rows represent the stepwise recombination maps. The results show that background selection makes the inferred time of population split more recent. The temporal change in rCCR in scenarios with high-recombining regions is even more affected with strong background selection.

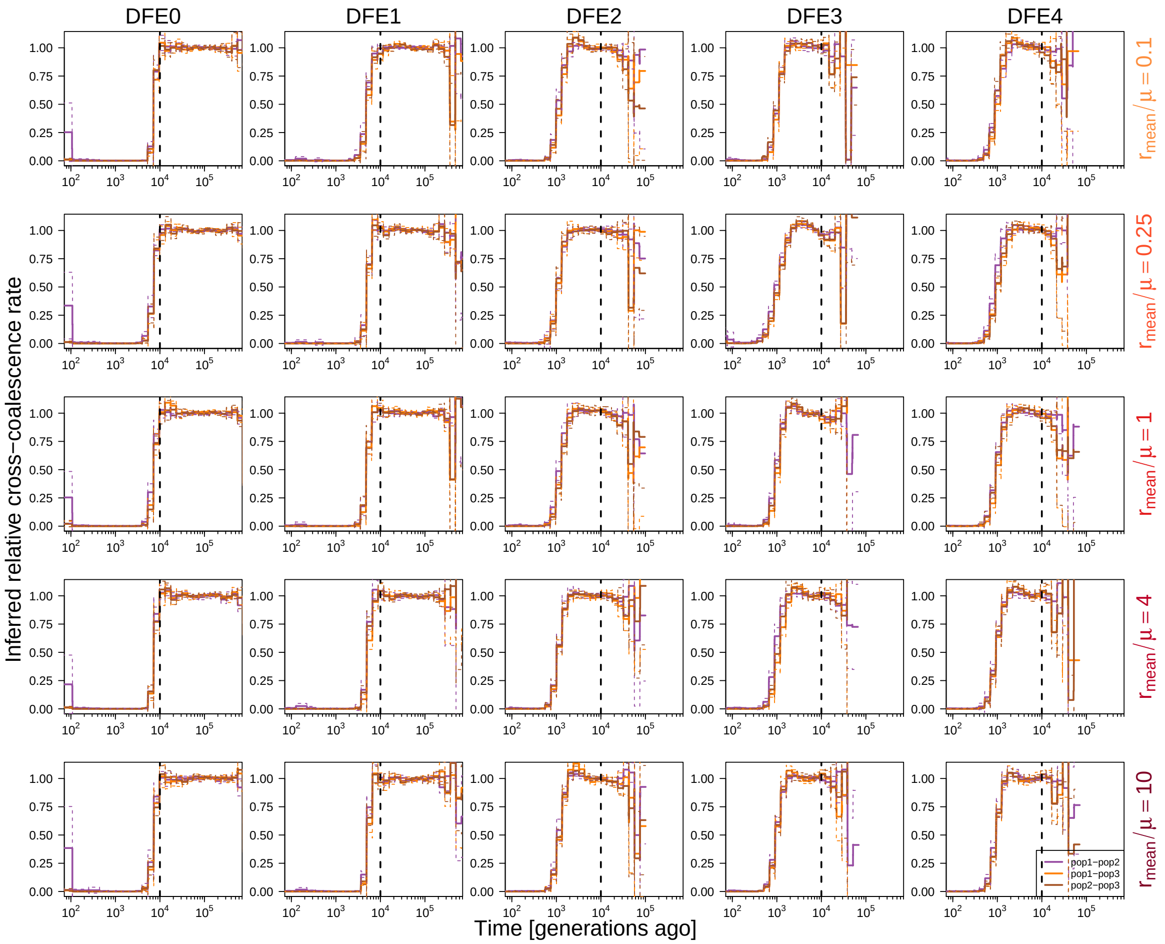

Figure S21: Inference of relative cross-coalescence rate by MSMC2 with background selection masking high-recombining regions. Columns represent different DFEs from neutral to strong background selection. Rows represent the stepwise recombination maps. While masking high-recombining regions eliminated the effect of high-recombining regions on the shape of rCCR trajectory, it does not remove the effect by background selection.

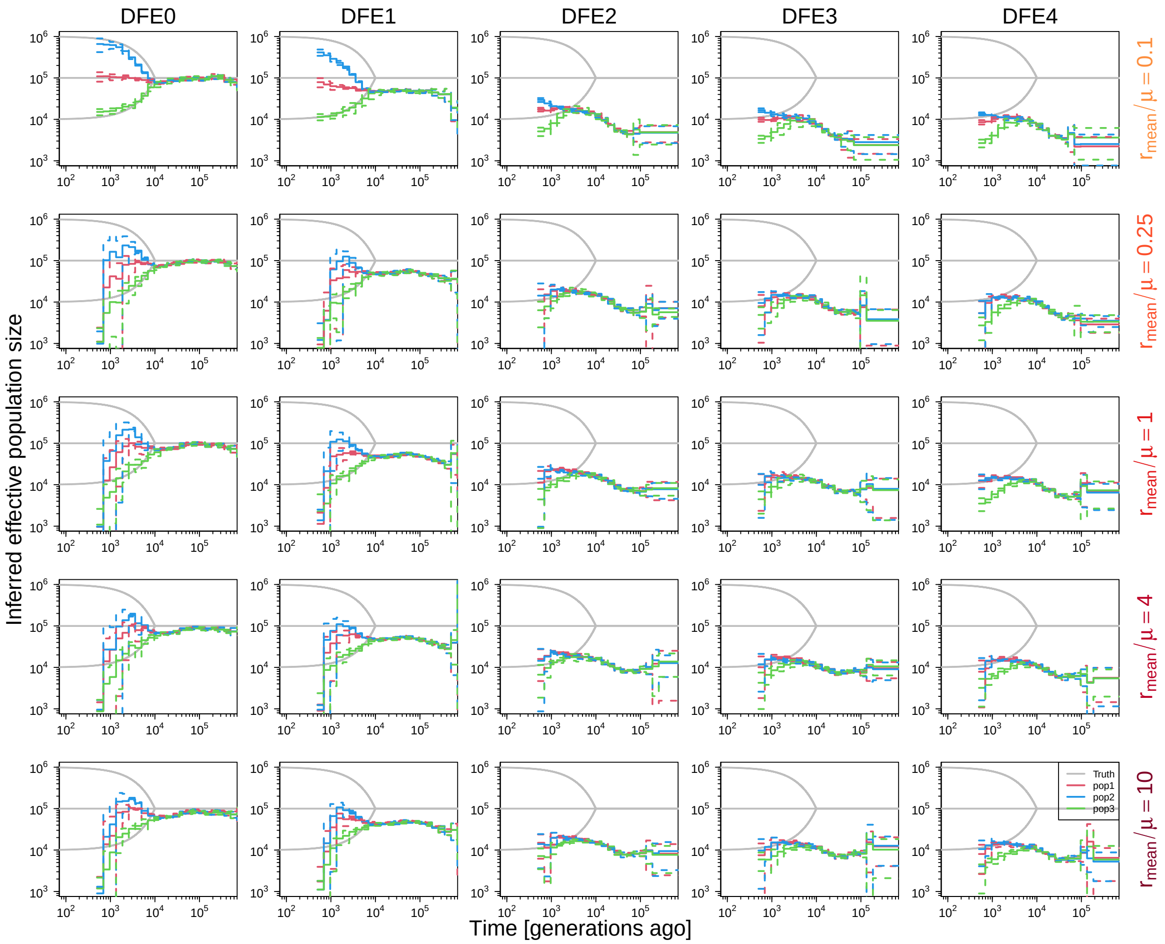

Figure S22: Inference of effective population size by `Relate` with background selection under control condition (not masking high-recombining regions). Columns represent different DFEs from neutral to strong background selection. Rows represent the stepwise recombination maps. The results show that background selection affects the scaling of inferred demography both in effective population size and time.

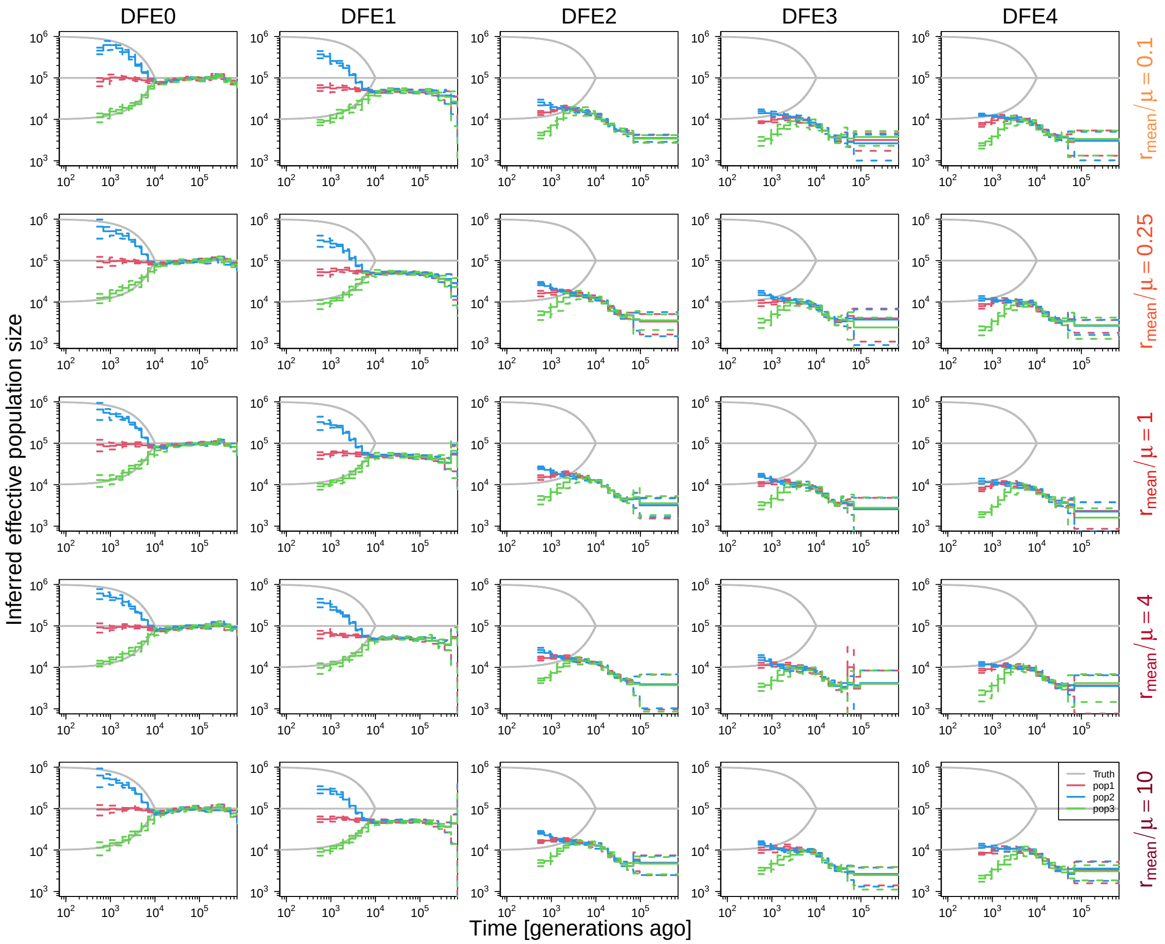

Figure S23: Inference of effective population size by `Relate` with background selection masking high-recombining regions. Columns represent different DFEs from neutral to strong background selection. Rows represent the stepwise recombination maps. The results show that while masking high-recombining regions eliminated the effect of high-recombining regions on the shape of demography trajectory in recent past, it does not remove the effect by background selection.

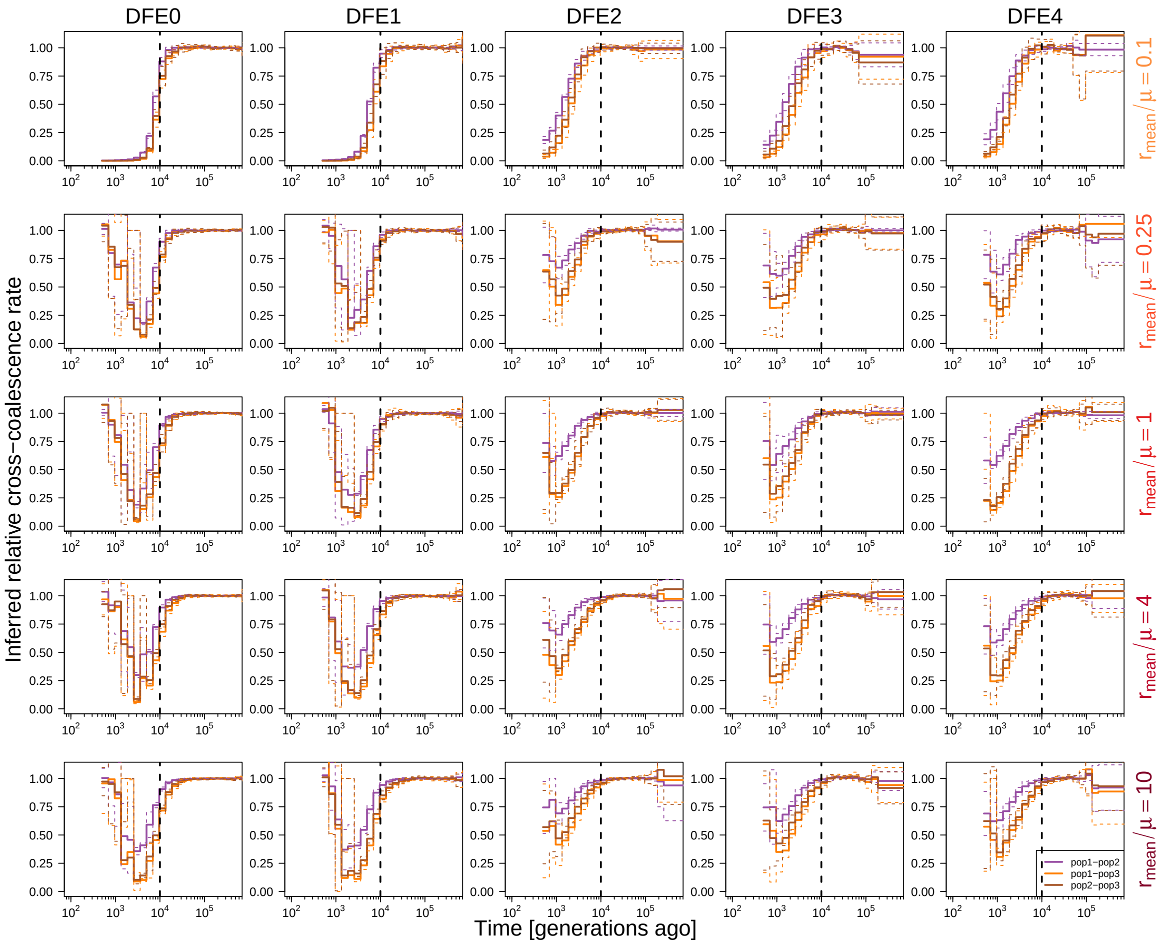

Figure S24: Inference of relative cross-coalescence rate by `Relate` with background selection under control condition (not masking high-recombining regions). Columns represent different DFEs from neutral to strong background selection. Rows represent the stepwise recombination maps. The results show that background selection makes the inferred time of population split more recent. The temporal change in rCCR in scenarios with high-recombining regions is even more affected with strong background selection.

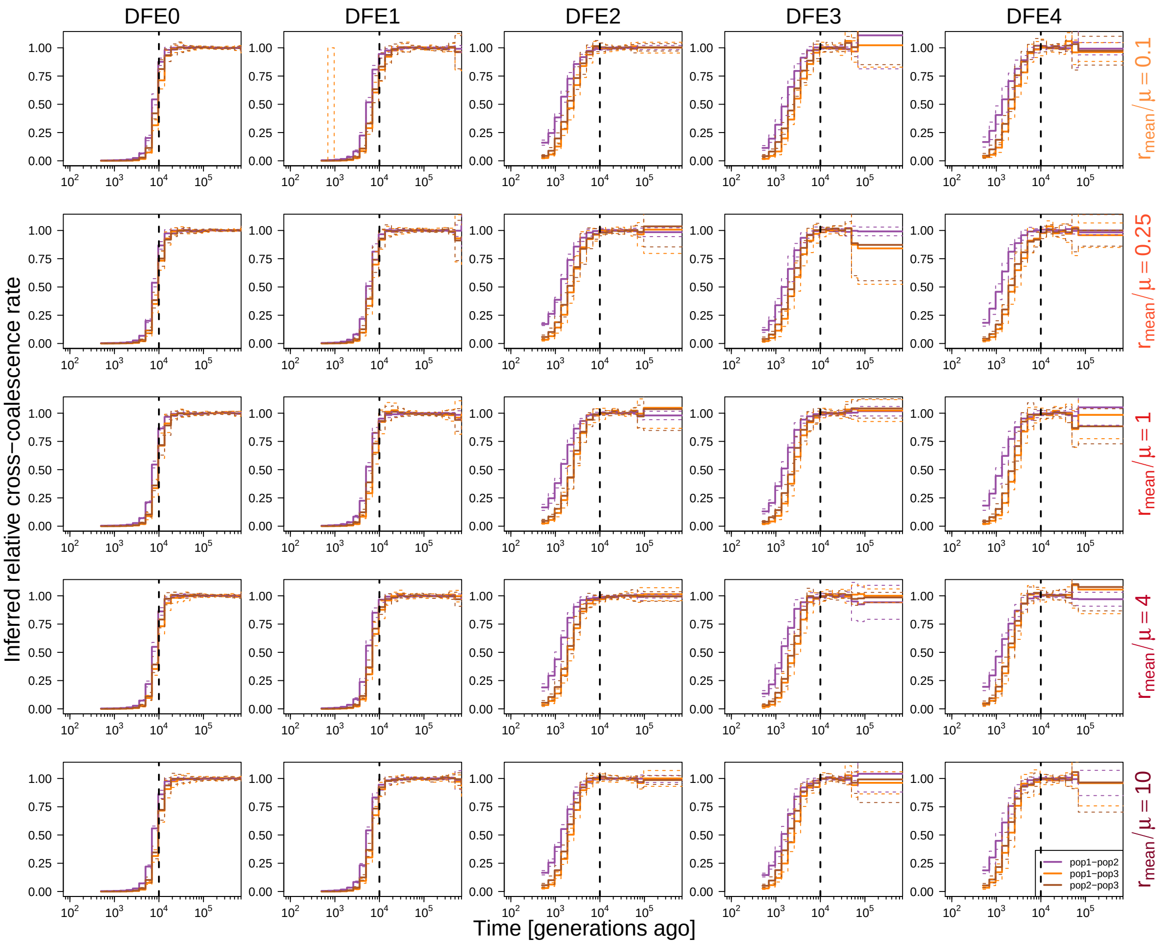

Figure S25: Inference of relative cross-coalescence rate by `Relate` with background selection masking high-recombining regions. Columns represent different DFEs from neutral to strong background selection. Rows represent the stepwise recombination maps. While masking high-recombining regions eliminated the effect of high-recombining regions on the shape of rCCR trajectory, it does not remove the effect by background selection.
